# X-ray diffraction: a powerful tool to probe and understand the structure of nanocrystalline calcium silicate hydrates

**DOI:** 10.1107/S2052519213021155

**Published:** 2013-09-19

**Authors:** Sylvain Grangeon, Francis Claret, Yannick Linard, Christophe Chiaberge

**Affiliations:** a BRGM, 3 avenue Claude Guillemin, Orléans, 45060, France; b ANDRA, Centre de Meuse/Haute Marne, 55290 Bure, France

**Keywords:** nanocrystalline calcium silicate hydrate, X-ray diffraction, interstratification, jennite, tobermorite

## Abstract

X-ray diffraction (XRD) patterns were calculated and compared to literature data with the aim of investigating the crystal structure of nanocrystalline calcium silicate hydrates (C-S-H), the main binding phase in hydrated Portland cement pastes. Published XRD patterns from C-S-H of Ca/Si ratios ranging from ∼ 0.6 to ∼ 1.7 are fully compatible with nanocrystalline and turbostratic tobermorite. Even at a ratio close or slightly higher than that of jennite (Ca/Si = 1.5) this latter mineral, which is required in some models to describe the structure of C-S-H, is not detected in the experimental XRD patterns. The 001 basal reflection from C-S-H, positioned at ∼ 13.5 Å when the C-S-H structural Ca/Si ratio is low (< 0.9), shifts towards smaller *d* values and sharpens with increasing Ca/Si ratio, to reach ∼ 11.2 Å when the Ca/Si ratio is higher than 1.5. Calculations indicate that the sharpening of the 001 reflection may be related to a crystallite size along c* (*i.e.* a mean number of stacked layers) increasing with the C-S-H Ca/Si ratio. Such an increase would contribute to the observed shift of the 001 reflection, but fails to quantitatively explain it. It is proposed that the observed shift could result from interstratification of at least two tobermorite-like layers, one having a high and the other a low Ca/Si ratio with a basal spacing of 11.3 and 14 Å, respectively.

## Abstract 2

The structure of nanocrystalline calcium silicate hydrates (C-S-H) was studied using X-ray diffraction and literature data. It is proposed that C-S-H of Ca/Si ratio ranging between ∼ 0.6 and ∼ 1.7 can be described as nanocrystalline tobermorite affected by turbostratic disorder. The broadening and shift of the basal reflection positioned between ∼ 13.5 and ∼ 11.2 Å (depending on the Ca/Si ratio) arises from nanocrystallinity and possibly from an interstratification phenomenon.

## 1. Introduction

Nanocrystalline calcium silicate hydrates (C-S-H), synthetic phases, are the major hydration products and the main binding phases in Portland cement (Richardson, 2008 ▶; Richardson *et al.*, 1994 ▶). Their chemical composition is variable. In particular, the structural calcium-to-silicon ratio (Ca/Si) is commonly assumed to vary from ∼ 0.6 to ∼ 2.3, the highest ratio being found in neat Portland cement, and lowest in cements containing products such as fly ash or metakaolin (Girão *et al.*, 2010 ▶; Richardson, 1999 ▶). As C-S-H control cement mechanical and chemical properties (Blanc *et al.*, 2010 ▶; Manzano *et al.*, 2007 ▶), they have been the subject of considerable amounts of work for decades (Richardson, 2008 ▶). Using a wide variety of physical methods (for example: NMR, IR, Raman and extended X-ray absorption fine structure spectroscopies, small-angle neutron scattering, X-ray diffraction, atomic pair distribution function or transmission electron microscopy), several research groups have investigated the C-S-H crystal structure (Allen & Thomas, 2007 ▶; Cong & Kirkpatrick, 1996*a* ▶,*b* ▶; Groves *et al.*, 1986 ▶; Kirkpatrick *et al.*, 1997 ▶; Lequeux *et al.*, 1999 ▶; Nonat, 2004 ▶; Rejmak *et al.*, 2012 ▶; Soyer-Uzun *et al.*, 2012 ▶; Yu *et al.*, 1999 ▶). All support the currently held view that C-S-H has a crystal structure close to tobermorite [Ca_4_Si_6_O_15_(OH)_2_·2H_2_O for the 11 Å variant studied by Merlino *et al.*, 1999 ▶, but the actual chemistry is variable] and/or jennite [Ca_9_Si_6_O_18_(OH)_6_·8H_2_O; Bonaccorsi *et al.*, 2004 ▶]. Tobermorite is usually thought to be a valid analogy for C-S-H of low Ca/Si ratio, whereas jennite structure, when required, is used to describe the structure of C-S-H of high Ca/Si ratio (Richardson, 2008 ▶; Taylor, 1986 ▶). Similarities between C-S-H and tobermorite have also been inferred from atomistic simulations (Pellenq *et al.*, 2009 ▶), although this model is subject to criticisms (Richardson, 2013 ▶).

**Figure 1:**
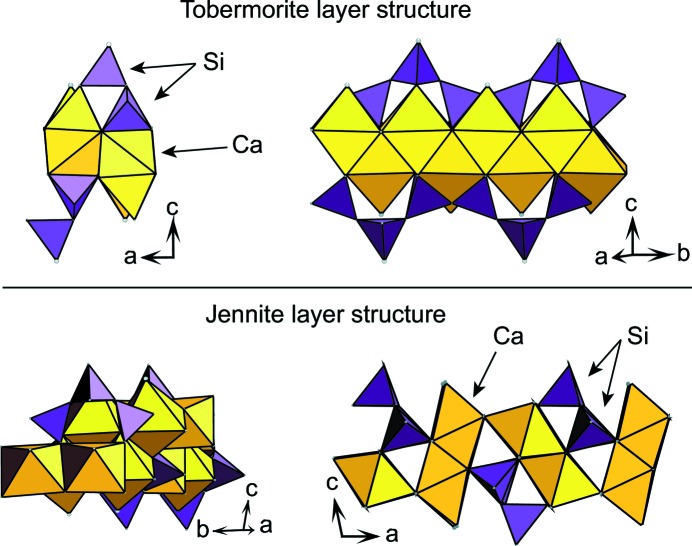
Tobermorite and jennite layer structures. Interlayer water and calcium are omitted for clarity.

Since X-ray diffraction (XRD) patterns from C-S-H exhibit only a few broad and weak diffraction maxima, this phase is often described as X-ray amorphous (Gartner *et al.*, 2000 ▶; Kirkpatrick *et al.*, 1997 ▶) or simply as amorphous (Mandaliev *et al.*, 2010 ▶). This latter adjective implies that C-S-H crystal structure would not have any long-range order. However, from literature data, C-S-H XRD patterns systematically have diffraction maxima at ∼ 7.4° 2θ Cu *K*α (12.0 Å), ∼ 16.7° 2θ Cu *K*α (5.3 Å), ∼ 29.1° 2θ Cu *K*α (3.1 Å), ∼ 32.0° 2θ Cu *K*α (2.8 Å), ∼ 49.8° 2θ Cu *K*α (1.8 Å), ∼ 55.0° 2θ Cu *K*α (1.7 Å) and ∼ 66.8° 2θ Cu *K*α (1.4 Å). These maxima are broad and mostly asymmetric, and the maximum at ∼ 7.4° 2θ Cu *K*α varies in position and intensity with C-S-H Ca/Si ratio (Garbev, Beuchle *et al.*, 2008 ▶; Garbev, Bornefeld* et al.*, 2008 ▶; Renaudin *et al.*, 2009 ▶). As electron diffraction patterns were also weakly modulated, Taylor (1986 ▶) concluded that such patterns could be attributable to either jennite or tobermorite, two minerals having a layered structure (Fig. 1 ▶) that exhibit diffraction maxima at ∼ 1.8 Å and in the 2.7–3.1 Å region. Thus, it can be inferred that C-S-H has a lamellar structure. By analogy with other layered structures (carbon black, layered silicates and manganates; Drits & Tchoubar, 1990 ▶; Grangeon *et al.*, 2010 ▶; Manceau *et al.*, 1997 ▶; Warren, 1941 ▶), line broadening is attributable to crystallite size in the nanometer range, whereas asymmetry is diagnostic of turbostratic disorder which is defined by the systematic occurrence, between successive layers, of random translations parallel to the layers and/or rotations about the normal, an assumption that has been recently validated by modeling of XRD patterns from four C-S-H samples having a Ca/Si ratio equal to ∼ 0.8 (Grangeon *et al.*, 2013 ▶). However, this previous study did not discuss the evolution of the C-S-H XRD pattern as a function of the Ca/Si ratio, and especially the variation in position of the maximum at ∼ 7.4° 2θ Cu *K*α, nor did it provide a calculated XRD pattern from nanocrystalline turbostratic jennite for comparison with nanocrystalline turbostratic tobermorite, and it may thus be wondered if XRD is accurate enough to distinguish between two highly defective lamellar structures of close chemistry and crystal structure. A positive answer would allow making a step further in deciphering the nature of C-S-H of various Ca/Si ratios.

The present study investigates C-S-H structure by means of XRD calculations, using a mathematical formalism dedicated to defective structure, with the aim of testing if there is any evidence for the presence of a jennite-like structure in C-S-H of high Ca/Si ratio and what is the origin of the shift of the reflection at ∼ 7.4° 2θ Cu *K*α when the C-S-H Ca/Si ratio varies.

## 2. Theoretical background

### 2.1. Software and structure models used for the calculations

All XRD calculations were performed using software developed by Plançon (1981 ▶, 2002 ▶), based on the mathematical formalism developed by Drits & Tchoubar (1990 ▶), and successfully applied to simulate XRD patterns from phyllosilicates, phyllomanganates and calcium silicate hydrates (Drits & Tchoubar, 1990 ▶; Grangeon *et al.*, 2008 ▶, 2013 ▶; Manceau *et al.*, 1997 ▶; Warren, 1941 ▶; Gaillot *et al.*, 2003 ▶; Lanson, Drits, Feng *et al.*, 2002 ▶; Lanson, Drits, Gaillot *et al.*, 2002 ▶). Unless otherwise stated, calculations involving tobermorite and jennite structures were performed using the structure models from, respectively, tobermorite MDO2 from Urals (Merlino *et al.*, 2001 ▶), and Bonaccorsi *et al.* (2004 ▶).

### 2.2. Calculation of *hk* diffraction bands

For a complete description of the mathematical formalism used for calculation of *hk* diffraction bands arising from turbostratic disorder, the reader is referred to Drits & Tchoubar (1990 ▶). Briefly, the intensity diffracted by a layered structure and normalized to a unit cell may be written as
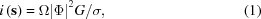
where *i* is the diffracted intensity, **s** is the vector characterizing the position of a point in reciprocal space [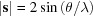, with θ and 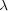 being the Bragg angle of diffraction and the wavelength of the radiation], 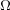 is the area of the unit-cell (equal to 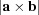, **a** and **b** being the in-layer plane unit cell axes), 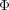 is the structure factor, *G* is the interference function and 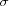 the area of the diffracting layer. The structure factor depicts the internal structure of the layer, whereas the interference function is a function of the stacking mode.

When random stacking faults are present in the crystal, the interference function may advantageously be written (Drits & Tchoubar, 1990 ▶; Méring, 1949 ▶) as
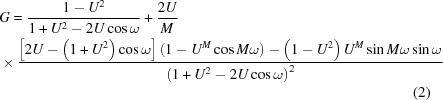
with
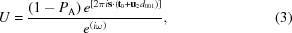
where 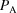 is the probability of finding a random stacking defect between two adjacent layers, **t**_0_ is the ordered translation, **u**_2_ is the unit vector along the normal to the layer (*i.e.* along c*), 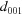 is the layer-to-layer distance, 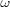 is (in a θ–2θ configuration) the angle between the normal perpendicular to **s** and the normal to the mean plane of the sample and *M* is the number of stacked layers.

When *P*_A_ = 0, the diffraction pattern exhibits the presence of *hkl* Bragg peaks. When *P*_A_ = 1 (*i.e.* systematic random stacking defect between adjacent layers), only *hk* diffraction bands are present. Still, *G* = 1, and the diffracted intensity remains a function of the structure factor. As such, the diffraction pattern contains all the information necessary to describe a unit cell (nature and position of the atoms; lattice constants). However, the XRD pattern from structures affected by turbostratic disorder significantly differs from that of a structure having three-dimensional order. In addition to the absence of the set of *hkl* reflections, replaced by *hk* bands (exclusion made of 00*l* reflections), diffraction maxima are shifted towards large angles compared with |*h* a* + *k* b*| (Warren, 1941 ▶), following the relation
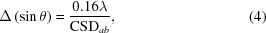
where CSD_*ab*_ (coherent scattering domain in the **ab** plane) size is the size of the crystallites in the layer plane. Consequently, *a* and *b* parameters cannot be determined from direct measurement of the position of the maxima.

### 2.3. Calculation of 00*l* reflections from interstratified structures

Interstratification is defined as the stacking along the c* axis of at least two types of layers of contrasting chemical or structural composition. Since the presently studied structures have turbostratic disorder, this type of structural arrangement only impacts basal (00*l*) reflections (see Ch. 3 in Drits & Tchoubar, 1990 ▶). In the present work, only interstratification of two different layer types is studied. Different interstratification cases may be differentiated from their degree of interaction between successive layers that is described by a factor called ‘Reichweite’ (Jagodzinski, 1949 ▶), and often written ‘*S*’. It is equal to the number of preceding layers which influence the probability of finding a layer at a given position. When *S* = 0, the stacking is fully random: the probability of finding a layer type at a certain position in the crystal does not depend on the types of preceding layers. When *S* = 1, the probability of finding a layer at a given position in the crystal depends on the nature of the preceding layer.

With *A* and *B* the two different layer types, and with the stacking mode following Markovian statistics, the structure of the interstratified particle is defined by the following parameters: the abundance of each of the two types of layers (*W_A_* and *W_B_*) and the junction probability from a layer type to the other (*P*_*AA*_, *P*_*BB*_, *P*_*AB*_, *P*_*BA*_, the latter being the probability that a layer of type *A* succeeds to a layer of type *B*).

When *S* = 0, then
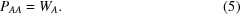
When *S* = 1, then the following relations apply
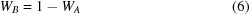


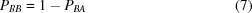


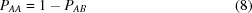


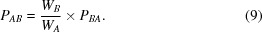
In the *S* = 1 configuration, different types of interstratification may be distinguished from the probability of succession of the layer type having the lowest abundance. If *W_A_* > *W_B_*, then the different interstratification types are distinguished by *P_BB_*. When *P_BB_* = *W_B_*, interstratification is random. When *P_BB_* = 1, then *P_AA_* = 1, which corresponds to a physical mixture of crystals, each containing only one layer type (*A* or *B*). In contrast, the maximum possible degree of ordering (MPDO) is reached when *P_BB_* = 0. Between random interstratification and MPDO is the partial ordering, whereas between random interstratification and segregation is the partial segregation.

The intensity diffracted by a crystal built from interstratified layers may be written as
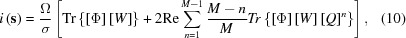
where Tr is the trace of the matrix, [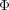] is the matrix containing the structure factors, [*W*] is the matrix containing the probability of occurrence from each layer type, and [*Q*] contains the junction probabilities.

As described by Méring (1949 ▶), one of the consequences of interstratification is that 00*l* reflections from mixed-layers containing *A* and *B* layer types will be positioned between 00*l* reflections of crystals built of solely *A* and solely *B* layers, with a breadth depending on the distance between 00*l* reflections of pure *A* and pure *B* crystals, and a position depending on the relative amounts of layers from type *A* and type *B* in the mixed-layered crystal (the higher the proportion of type *A* layers, the closer the position to that of a crystal built of type *A* layers).

For more details, the reader is referred to previous studies (Drits & Tchoubar, 1990 ▶; Lanson, 2011 ▶; Lanson *et al.*, 2009 ▶; Claret *et al.*, 2004 ▶).

## 3. Description of C-S-H layer structure

### 3.1. Origin of the diffraction maxima in the high-angle region (15–80° 2θ Cu *K*α: 5.9–1.2 Å) of C-S-H XRD patterns

**Figure 2:**
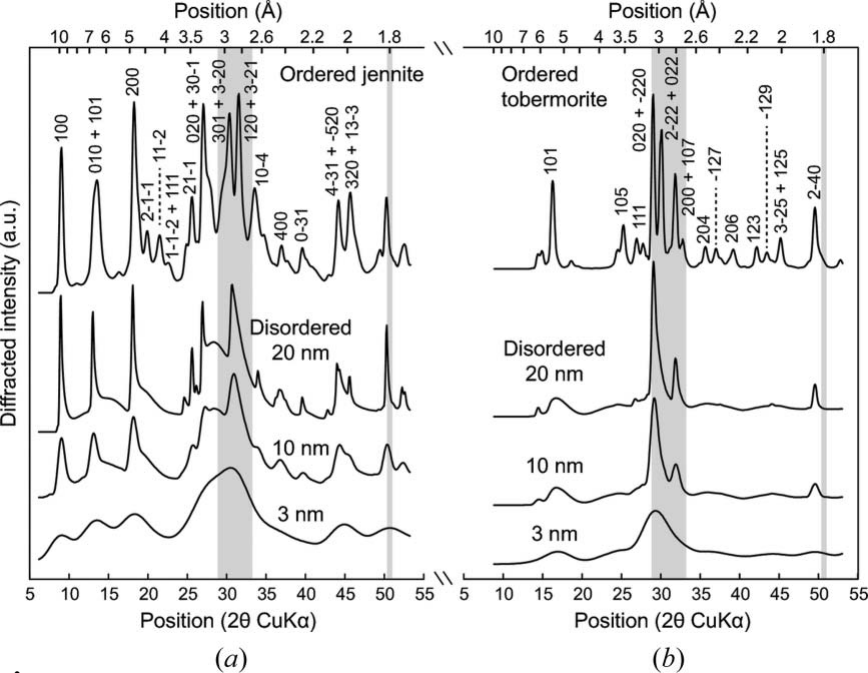
Calculated XRD patterns of (*a*) jennite and (*b*) tobermorite. Indexing (non-exhaustive) was performed using models from Bonaccorsi *et al.* (2004 ▶) and Merlino *et al.* (2001 ▶). Basal reflections are omitted. For each mineral, the theoretical XRD patterns (from top to bottom) of an ordered form having a mean CSD_ab_ size of 20 nm, and from disordered variations having a CSD_ab_ size of 20, 10 and 3 nm are presented. Grey areas correspond to the regions where Taylor (1986 ▶) identified the overlapping tobermorite and jennite diffraction maxima.

Jennite and tobermorite calculated XRD patterns are presented in Fig. 2 ▶. Basal reflections, which are not expressed in the high-angle region of published C-S-H XRD patterns, are not calculated during this step but are discussed hereafter, as literature data indicate a specific behavior when C-S-H Ca/Si ratio varies. Both an ordered form (*i.e.* without stacking faults) having a mean CSD_*ab*_ size of 20 nm and a turbostratic variation (hereafter referred as ‘disordered’) were calculated for jennite and for tobermorite. The effect of crystallite size was investigated for the disordered variation by calculating the XRD patterns of crystallites having CSD_*ab*_ sizes of 20, 10 and 3 nm. Calculated XRD patterns from ordered minerals compare well with experimental XRD patterns (Manzano *et al.*, 2010 ▶). In disordered variations, only asymmetric *hk* bands are expressed; decreased CSD_*ab*_ size weakens and broadens these diffraction maxima, the weakest becoming invisible (Fig. 2 ▶).

As discussed by Taylor (1986 ▶), XRD patterns from ordered jennite and tobermorite have maxima at ∼ 1.8 Å (50.7° 2θ Cu *K*α) and in the 2.7–3.1 Å (28.8–33.2° 2θ Cu *K*α) region (Fig. 2 ▶). This holds true for the disordered variations, whatever the CSD_*ab*_ size, so the sole identification of these features cannot be used to distinguish between tobermorite and jennite. However, the present calculations allow proposing an indicator, independent of the crystallinity (ordered or disordered), to discriminate between jennite and tobermorite. In the 10–4 Å range (9–22° 2θ Cu *K*α), jennite has three intense diffraction maxima at ∼ 9.8 Å (9.0° 2θ Cu *K*α), ∼ 6.8 Å (13.0° 2θ Cu *K*α) and ∼ 4.9 Å (18.1° 2θ Cu *K*α). The maximum at ∼ 4.9 Å is partially overlapped by a diffraction maximum in the disordered version of tobermorite (Fig. 2 ▶). Additionally, portlandite, often observed as an impurity of C-S-H synthesis (*e.g.* Garbev, Beuchle *et al.*, 2008 ▶; Renaudin *et al.*, 2009 ▶), has an intense Bragg peak at 4.8 Å (18.5° 2θ Cu *K*α). Thus, the maximum at ∼ 4.9 Å cannot be easily used to discriminate between jennite and tobermorite. Similarly, the maximum at ∼ 9.8 Å from the jennite XRD pattern is at an intermediate position between the basal reflections of the 9 Å and the 11 Å variations of tobermorite (Merlino *et al.*, 1999 ▶), and may be mistaken. Such potential overlaps were not identified for the diffraction maximum at ∼ 6.8 Å, which is suggested here as an indicator to distinguish between tobermorite and jennite XRD patterns.

From qualitative comparison with C-S-H XRD patterns available in the literature, C-S-H certainly has a disordered layered structure similar to tobermorite: none of the published XRD patterns exhibit a diffraction maxima at ∼ 6.8 Å; however, two diffraction maxima at 3.05 and 2.80 Å, with a relative intensity of ∼ 1/4, as well as a weak and asymmetrical maximum at 5.5 Å, are systematically present. All of the features expressed in the XRD patterns from C-S-H are successfully reproduced in the calculation of a disordered tobermorite structure with a CSD_*ab*_ size of 20–10 nm (Fig. 2 ▶). Thus, C-S-H certainly has a tobermorite-like structure affected by turbostratic disorder, at least when the structural Ca/Si ratio ranges between ∼ 0.6 and ∼ 1.7 (Garbev, Beuchle *et al.*, 2008 ▶; Renaudin *et al.*, 2009 ▶). The actual lowest Ca/Si ratio that C-S-H can accommodate is uncertain, because of the presence of amorphous silica when Ca/Si is lower than ∼ 0.6 (Garbev, Bornefeld *et al.*, 2008 ▶). Similarly, at high Ca/Si ratio (≥ 1.7), the presence of Bragg peaks from portlandite (Ca(OH)_2_) is almost systematically observed and the structural Ca/Si ratio from corresponding C-S-H samples cannot be reliably estimated. To the author’s knowledge, only Chen *et al.* (2004 ▶) reported the XRD pattern of a portlandite-free C-S-H with Ca/Si equal to 1.7, which is fully compatible with turbostratic tobermorite.

### 3.2. Implications for the models of C-S-H structure

In his comprehensive review, Richardson (2008 ▶) summarized the different models that aim at describing the evolution of C-S-H structure as a function of Ca/Si ratio. The main discrepancy between the different models lies in the representation of C-S-H structure at high Ca/Si ratios (> ∼ 1.5). In particular, the model from Taylor (1986 ▶) assumes the presence of jennite-like units. However, from the present literature survey, there is no evidence from XRD for the presence of jennite in published C-S-H XRD patterns, except the laboratory-synthesized sample D69 from Brunauer & Greenberg (1960 ▶) which exhibited diffraction maxima compatible with ordered jennite (Gard & Taylor, 1976 ▶). Although the original synthesis procedure has been reported, Taylor (1997 ▶) stated that ‘attempts to repeat these preparations […] have failed, and the conditions under which the product is formed are obscure’. It is thus unclear how representative this sample is of a product that can actually be found in hydrated pastes, and this questions the validity of the model from Taylor (1986 ▶). Acquisition of XRD patterns from portlandite-free C-S-H samples having a Ca/Si ratio ≥ 1.7 would be mandatory to gain better insight into the validity of this model. Nevertheless, from the proposed calculations it is clear that for a Ca/Si ratio ranging between ∼ 0.6 and ∼ 1.7 solely tobermorite is needed to describe C-S-H crystal structure, even for a Ca/Si ratio close to jennite. The almost systematic presence of portlandite in samples having a high Ca/Si ratio (≥ 1.7) may indicate that the tobermorite/CH (CH standing for calcium hydroxide) model from Richardson most adequately describes the evolution of C-S-H structure as a function of Ca/Si ratio.

## 4. Description of layer stacking

**Figure 3:**
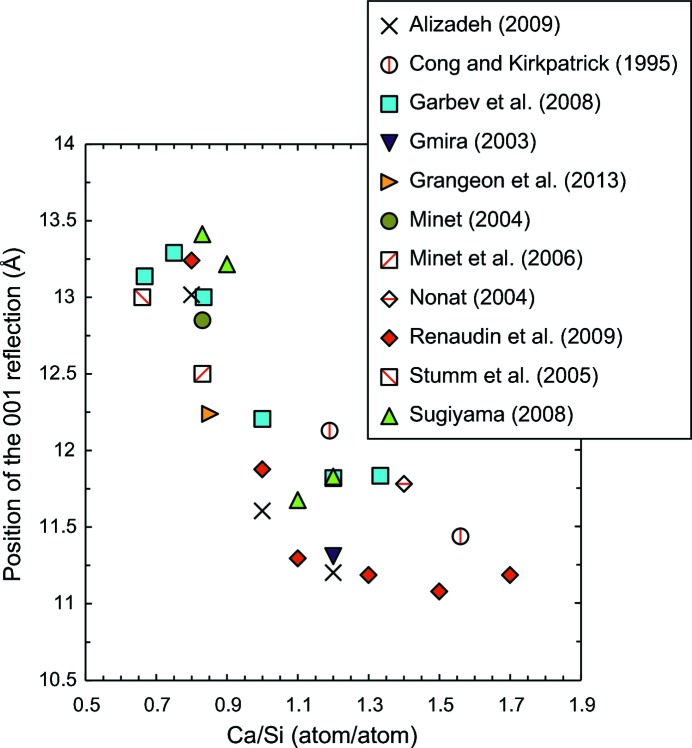
Variation in the position of the C-S-H basal reflection as a function of structural Ca/Si ratio. Published XRD patterns having too weak a diffraction intensity or showing the presence of high amounts of portlandite were excluded. Note that the actual Ca/Si ratio may be slightly overestimated for samples from Renaudin *et al.* (2009 ▶) that have Ca/Si > 1.5, because of the presence of portlandite in their XRD pattern.

At least two research groups have conducted insightful discussion on the evolution of C-S-H XRD patterns as a function of the C-S-H Ca/Si ratio (Garbev, Beuchle *et al.*, 2008 ▶; Renaudin *et al.*, 2009 ▶). In these articles the diffraction maximum exhibiting the strongest variation in position is the reflection between ∼ 13.5 and ∼ 11.2 Å (6.5–7.9° 2θ Cu *K*α), which corresponds to the C-S-H 001 reflection, *i.e.* to the layer-to-layer distance (Grangeon *et al.*, 2013 ▶). Data from Garbev, Beuchle *et al.* (2008 ▶) and Renaudin *et al.* (2009 ▶) compare well with other published data (Alizadeh, 2009 ▶; Cong & Kirkpatrick, 1995 ▶; Gmira, 2003 ▶; Grangeon *et al.*, 2013 ▶; Minet, 2004 ▶; Minet *et al.*, 2006 ▶; Nonat & Lecoq, 1996 ▶; Stumm *et al.*, 2005 ▶; Sugiyama, 2008 ▶), as illustrated in Fig. 3 ▶. At low Ca/Si ratio (< 0.9), the basal distance is close to 13.5 Å. With increasing Ca/Si ratio, this distance reduces down to ∼ 11.2 Å for Ca/Si = 1.7.

**Figure 4:**
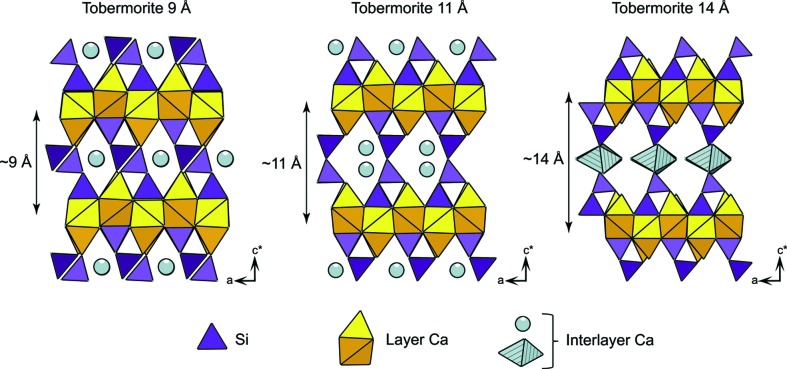
Schematic view of the three main variations of tobermorite, as defined by their layer-to-layer distance, equal to (from left to right): 9, 11 and 14 Å. Interlayer water molecules omitted for clarity in 9 and 11 Å variations, but represented in the coordination sphere of interlayer Ca in the 14 Å variation to highlight layer-to-layer connectivity.

Note that when C-S-H of the Ca/Si ratio = 1.19 is heated at 473 K for 3 h, the basal distance further reduces to 9.6 Å (Cong & Kirkpatrick, 1995 ▶). The ∼ 11 and 14 Å basal distances are also observed in natural tobermorite (Bonaccorsi *et al.*, 2005 ▶; Merlino *et al.*, 2001 ▶), and a ∼ 11 Å variant has also been observed to collapse to 9.6 Å upon heating at 498 K for 3 h (Merlino *et al.*, 1999 ▶). The 14, 11 and 9 Å distances for tobermorite are schematized in Fig. 4 ▶; because these three basal distances are also observed in C-S-H, the assumption of structural similarity between C-S-H and tobermorite is reinforced.

There are at least three main phenomena which may be used to explain the shift, at room temperature, of the basal reflection from ∼ 11.2 Å to ∼ 13.5 Å with decreasing Ca/Si ratio. The first, suggested by Garbev and coworkers (Garbev, Beuchle *et al.*, 2008 ▶; Garbev, Bornefeld* et al.*, 2008 ▶), is that with increasing Ca/Si ratio the site occupancy of bridging Si in wollastonite-like chains decrease, which decreases the occupancy of the interlayer space, thus allowing a reduction of the layer-to-layer distance through the action of interlayer calcium bridging two adjacent layers. For more details, the reader is referred to Fig. 8 from Garbev, Bornefeld *et al.* (2008 ▶).

**Figure 5:**
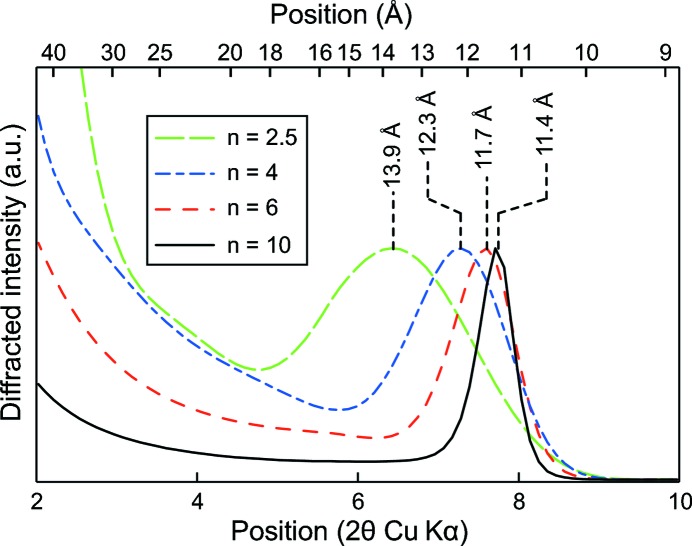
Theoretical intensity and position from the basal reflection of a sample built up of 11 Å tobermorite having a mean number of *n* = 10 (solid line), 6 (dashed line), 4 (dashed-dotted line) and 2.5 (long-dashed line) stacked layers. Distribution is log-normal.

The second phenomenon is a decrease in the crystallite size along c*, *i.e.* a decrease in the mean number of stacked layers. Such a decrease leads to a shift of the 001 reflection towards the low-angle region is exacerbated for particles such as C-S-H having sizes in the nanometer range (*e.g.* Skinner *et al.*, 2010 ▶; Grangeon *et al.*, 2013 ▶), and it has already been observed in other lamellar structures (Drits & Tchoubar, 1990 ▶; Grangeon *et al.*, 2012 ▶; Lanson *et al.*, 2008 ▶). It mainly originates from the fact that in the low-angle region of XRD patterns, the Lorentz polarization factor is strongly rising towards the low-angle part and thus this artificially displaces the diffraction maxima (Reynolds, 1968 ▶, 1986 ▶), although other minor effects are also involved (*e.g.* Truntz, 1976 ▶). This is illustrated in Fig. 5 ▶, taking tobermorite as a model structure. The deviation from the theoretical position is as large as 2.5 Å when the mean number of stacked layers is reduced from 10 to 2.5. The shift towards low angles is accompanied by a broadening of the Bragg peak. Thus, if this phenomenon was responsible for the shift observed in the literature data (Fig. 3 ▶), an increase in the FWHM of the 001 reflection should be observed when the Ca/Si ratio decreases. FWHM was measured by Garbev, Beuchle *et al.* (2008 ▶), who observed an increase of this parameter from 0.72° to ∼ 0.85–1.5° when the Ca/Si ratio decreased from 1.33 to 0.66–0.75. A FWHM of 0.72° is close, in Fig. 5 ▶, to the calculation performed with a mean number of 10 stacked layers (FWHM = 0.61°). Assuming that the shift of the 001 reflection is only due to a decrease in the mean number of stacked layers with decreasing Ca/Si ratio and using Fig. 5 ▶, the ∼ 2.3 Å shift (from ∼ 11.2 to ∼ 13.5 Å) would mean that at low Ca/Si, the mean number of stacked layers is equal to ∼ 2.5 and the FWHM is 1.81°, about twice the FWHM measured by Garbev, Beuchle *et al.* (2008 ▶) for C-S-H samples with low Ca/Si (0.85–1.05°). Thus, even if a decrease in the mean number of layers stacked coherently with decreasing structural Ca/Si contributes to the observed shift of the 001 reflection (Grangeon *et al.*, 2013 ▶), it cannot quantitatively account for the magnitude of the shift observed by Garbev and coworkers, and another hypothesis has to be formulated. This conclusion is obvious when looking at data from Renaudin *et al.* (2009 ▶) as, from their Fig. 3 ▶, the FWHM does not evolve significantly with the C-S-H Ca/Si ratio. Note, however, that variation in the mean number of stacked layers certainly explains the data dispersion observed in Fig. 3 ▶ (at a similar Ca/Si ratio different samples can have a different mean number of stacked layers, leading to data scattering along the *y* axis), although an effect of the synthesis protocol and of sample preservation may be a factor.

**Figure 6:**
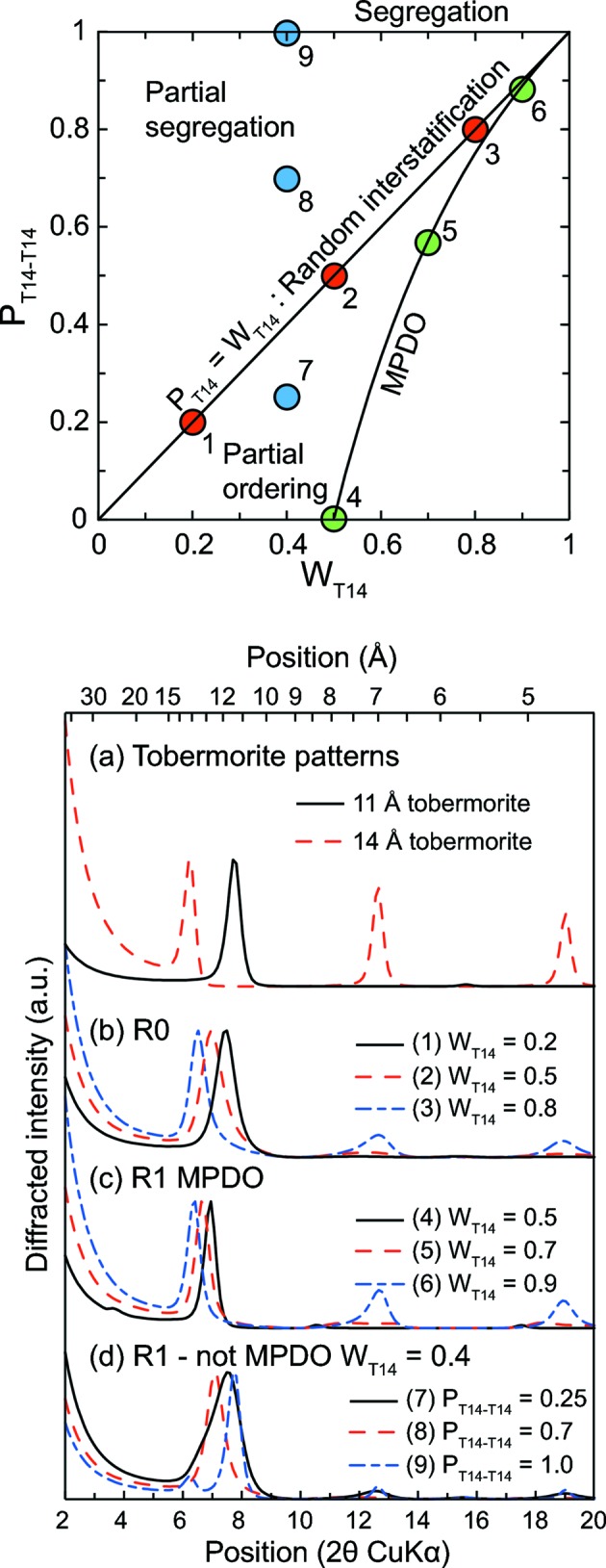
Impact of interstratification on the 00*l* reflections from a theoretical mixed-layer tobermorite XRD pattern. (top) Different interstratification modes for a binary system containing tobermorite-like layers having 14 and 11 Å basal distances, plotted in a *P*_T14-T14_ *versus W*_T14_ graph. The structure of the 14 Å layer is that of plombierite, and the structure of the 11 Å layer is that from a MDO2 sample from Urals where all bridging tetrahedra are omitted. Solid line: field of R1 maximum possible degree of order (R1 MDPO), dotted line: field of R0 (random interstratification). Between these two lines lies the field of partial ordering, and above the R0 field lies the partial segregation field. Segregation is reached when *P*_T14-T14_ = 1. The area below the MPDO field is a non-physical field. Dots labelled 1–9 represent the nine calculations performed in the bottom figure: 1–3 are calculations performed in the R0 hypothesis (*b* in the bottom figure, with *W*_T14_ = 0.2, 0.5 and 0.8 for calculations 1 – solid line, 2 – dashed line and 3 – dash-dotted line), 4–6 calculations in the R1 MPDO hypothesis (*c* in the bottom figure, *W*_T14_ = 0.5, 0.7 and 0.9 for 4 – solid line, 5 – dashed line and 6 – dash–dotted line) and 7–9 calculations performed in the R1 non-MDPO hypothesis (*d* in the bottom figure, *W*_T14_ = 0.4 and *P*_T14-T14_ equal to 0.2, 0.5 and 1 for 7 – solid line, 8 – dashed line and 9 – dash–dotted line); (*a*) in the bottom figure presents the theoretical basal reflections from pure 11 and 14 Å tobermorite (solid and dashed lines, respectively).

**Figure 7:**
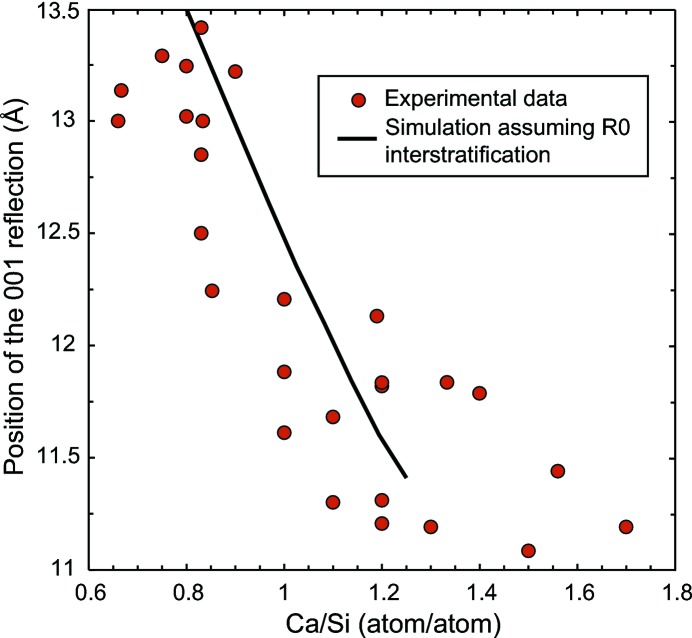
Evolution of the position from the 001 reflection in experimental data (dots, same data as in Fig. 3 ▶) and in a hypothetical random (R0) interstratified structure (solid line) built of layers of plombierite-like layers [*d*(001) = 14 Å] with interlayer calcium omitted yielding a Ca/Si ratio of 2/3 and of layers of tobermorite MDO2 from Urals [*d*(001) = 11.3 Å] with the occupancy of interlayer calcium (Ca2 in Merlino *et al.*, 2001 ▶) increased to 0.5 and all Si bridging tetrahedra omitted, yielding a Ca/Si ratio of 5/4.

The third hypothesis that may explain the shift of the 001 reflection as a function of Ca/Si ratio is the interstratification from a layer of low Ca/Si ratio with one of high Ca/Si ratio, the first being dominant for C-S-H of low structural Ca/Si ratio, and *vice versa*. To illustrate this effect, calculations were performed using a theoretical 14 Å tobermorite (plombierite) with interlayer calcium omitted as the low Ca/Si end-member, and a theoretical 11 Å tobermorite MDO2 from Ural with all bridging Si tetrahedra omitted as the high Ca/Si end-member. All main interstratification hypotheses were tested (Fig. 6 ▶): random interstratification of 11 and 14 Å layers (R0 interstratification), R1 interstratification and interstratification with the maximum possible degree of ordering (R1-MPDO; *P*_T14-T14_ = 0 if *W*_T14_ ≤ 0.5). In the R0 case, the shift of the 001 reflection towards 14 Å with decreasing Ca/Si ratio is accompanied by an increase of the FWHM compared with end-members, which is not the case in the R1-MPDO hypothesis, where the 001 reflection remains very sharp. Note the presence of a reflection at ∼ 25 Å in the R1-MPDO case, when W_T14_ = 0.5. This superstructure results from the regular alternation of 11 and 14 Å layers. In the R1 (non-MPDO) hypothesis, the 001 reflection is highly asymmetrical when the calculations lie in the partial order range, and two peaks, corresponding to the 001 reflection from 11 and 14 Å species, are resolved when the calculations lie in the segregation range. If the observed shift from the 001 reflection results from interstratification, then it most likely is a R0 interstratification, because in the R1 hypothesis peaks are too sharp (and when *W*_T14_ = 0.5, a maximum at ∼ 25 Å is present), and in the R1-MPDO hypothesis the 001 is either too asymmetric or split into two maxima occurring at ∼ 11 and 14 Å. Such a hypothesis is reinforced with the good agreement obtained between the evolution of the calculated position from the maximum of the 001 reflection, assuming a R0 interstratification, and literature data (Fig. 7 ▶). Note that using the structural model of tobermorite MDO2 from Urals, removing all bridging tetrahedra and increasing the occupancy of interlayer calcium to the highest possible value (according to Merlino *et al.*, 2001 ▶) did not achieve a Ca/Si ratio higher than 5/4. This may indicate that C-S-H of a higher ratio have an additional crystallographic site for calcium and/or that Si monomers are present. The R0 interstratification has also been invoked by Taylor and coworkers (Heller & Taylor, 1956 ▶; Taylor & Howison, 1956 ▶). Finally, the 11 Å layer used for this calculation, in which all bridging Si tetrahedra are omitted and which contains interlayer Ca, presents some structural similarity with a 11 Å layer, with all Si bridging tetrahedra omitted, sandwiched between Ca(OH)_2_ sheets, which is compatible with the T/CH model from Richardson which assumes that tobermorite layers are interstratified with Ca(OH)_2_ sheets (Girão *et al.*, 2010 ▶).

## 5. Summary

Despite only a few broad diffraction maxima in C-S-H XRD patterns, meaningful and accurate structural information can be extracted. The present study indicates C-S-H has a structure close to tobermorite, whatever the Ca/Si ratio. A jennite component does not need to be invoked to explain the experimental XRD patterns. This does not mean, however, that a jennite-like structure does not exist at Ca/Si ≥ ∼ 1.7, but rather that more XRD data from samples having these ratios must be acquired.

Variation in the position of the 001 reflection, from ∼ 13.5 Å at low Ca/Si to ∼ 11.2 Å at high Ca/Si ratio, cannot be quantitatively accounted for by an increase in crystallite size along c* with increasing Ca/Si ratio, although it does certainly contribute. In addition to the mechanism proposed by Garbev and coworkers (Garbev, Beuchle *et al.*, 2008 ▶; Garbev, Bornefeld *et al.*, 2008 ▶), the random interstratification of plombierite-like layers (having a low Ca/Si), with 11 Å tobermorite in which all Si bridging tetrahedra are omitted (having a high Ca/Si ratio) is proposed. Further work is required to check the validity of this assumption. Indeed, the present hypothesis is based solely on the examination of the 001 reflection of C-S-H XRD patterns whereas, as discussed by Claret *et al.* (2004 ▶), one should consider with caution the use of a single peak variation to draw conclusions. For clays, a multispecimen method is mandatory (Ferrage *et al.*, 2005 ▶; Lanson *et al.*, 2009 ▶; Sakharov *et al.*, 1999 ▶). In order to be transposed to C-S-H, this would first require acquiring XRD patterns from an oriented preparation to check the rationality of 00*l* reflections. Although such an experimental set-up is common in clay science, from preliminary tests performed in our research group, an oriented preparation will be much more difficult to obtain in the case of C-S-H because: (i) particle anisotropy is much less important than that from clays, (ii) C-S-H crystals are hardly dispersed, probably as a result of a very high surface charge, (iii) preparation cannot be performed in water because of the fast equilibrium between C-S-H and water leading to partial sample dissolution, and (iv) contact with the atmosphere must be avoided, or C-S-H suffer from carbonation.
